# Urokinase-type plasminogen activator receptor (uPAR), tissue factor (TF) and epidermal growth factor receptor (EGFR): tumor expression patterns and prognostic value in oral cancer

**DOI:** 10.1186/s12885-017-3563-3

**Published:** 2017-08-25

**Authors:** Anders Christensen, Katalin Kiss, Giedrius Lelkaitis, Karina Juhl, Morten Persson, Birgitte Wittenborg Charabi, Jann Mortensen, Julie Lyng Forman, Anne Lyngholm Sørensen, David Hebbelstrup Jensen, Andreas Kjaer, Christian von Buchwald

**Affiliations:** 1 Department of Otolaryngology, Head & Neck Surgery and Audiology, Rigshospitalet, University of Copenhagen, Copenhagen, Denmark; 2 Department of Clinical Physiology, Nuclear Medicine & PET and Cluster for Molecular Imaging, Rigshospitalet, University of Copenhagen, Copenhagen, Denmark; 3 Department of Pathology, Rigshospitalet, University of Copenhagen, Copenhagen, Denmark; 4 Department of Public Health, Section of Biostatistics, University of Copenhagen, Copenhagen, Denmark

**Keywords:** Oral squamous cell carcinoma, uPAR, Tissue factor, EGFR, prognosis, immunohistochemistry, margins, oral cancer, molecular imaging

## Abstract

**Background:**

Tumor-specific biomarkers are a prerequisite for the development of targeted imaging and therapy in oral squamous cell carcinoma (OSCC). urokinase-type Plasminogen Activator Receptor (uPAR), Tissue Factor (TF) and Epidermal Growth Factor Receptor (EGFR) are three biomarkers that exhibit enhanced expression in many types of cancers, and have been investigated as potential biomarkers for targeted strategies and prognostication. The aim of the study was to investigate the expression patterns of uPAR, TF and EGFR and their potential prognostic value in OSCC.

**Methods:**

Immunohistochemical expression of uPAR, TF and EGFR in tumor resection specimens from 191 patients with primary OSCC was analyzed. Overall (OS) and disease-free survival (DFS) was calculated. Associations between biomarker expression, clinicopathological factors and patient survival was analyzed using the Cox proportional hazards model for univariate and multivariate analysis, log rank and Kaplan-Meier statistics.

**Results:**

uPAR and TF exhibited a highly tumor-specific expression pattern while EGFR also showed expression in normal tissues outside the tumor compartment. The overall positive expression rate of uPAR, TF and EGFR was 95%, 58% and 98%, respectively. High uPAR expression across the entire cohort was negatively associated with OS (*p* = 0.031, HR = 1.595 (95%CI 1.044–2.439)) in univariate analysis. The 5-year OS for high and low uPAR expression was 39% and 56%, respectively. The expression of TF and EGFR was not associated with survival outcome.

**Conclusions:**

This study may suggest that uPAR and TF could potentially be attractive targets for molecular imaging and therapy in OSCC due to high positive expression rates and tumor-specific expression patterns. High uPAR expression was significantly associated with a reduced survival. uPAR seems to be a prognostic biomarker in oral cancer.

## Background

Oral cavity cancer is the 11th most common cancer worldwide and accounts for substantial mortality and morbidity for individuals affected by this disease [1]. Despite important technological advances in diagnosis and therapy especially in the last decades, the prognosis for OSCC has only moderately improved, and reported overall survival rate has remained at roughly 50% [2]. Surgery is a cornerstone in the treatment of primary OSCC with curative intent, whether the objective is to achieve complete removal of the tumor as well as any regional metastatic disease in the neck. Failure to achieve a clear tumor-resection margin, and to detect residual disease in the surgical bed intraoperatively, has direct major negative impact on the chances for cure not fully compensated for by adjuvant radiotherapy [3, 4]. Intraoperative detection and delineation of cancer is still based on visual inspection and palpation of the tissues obviating reliable assessment of the microscopic extent of the disease. Consequently, non-radical surgery remains a major challenge, and novel imaging technology, that enables accurate planning of surgery and intraoperative tumor detection, is warranted.

The discovery of a large number of tumor-specific biomarkers has stimulated new optimism in the development of targeted imaging and treatment of cancer [5]. Ideally, a biomarker suitable for targeting purposes should have strong expression within the tumor compartment, and absent or insignificant expression in adjacent normal tissue. The expression of a specific biomarker may vary between different types of cancer, and within each specific type of cancer due to tumor heterogeneity, and therefore studies designed to examine the exact histological expression and tumor-specificity of different targets in large patient cohorts, are becoming increasingly important. Furthermore, accumulating evidence has validated biomarker expression and profiling as an important tool for individual risk stratification and planning of patient-tailored treatment [6]. The combined use of a specific biomarker as a prognosticator and a tumor-specific target for theranostic purposes is a novel strategy, which may have potential applications in the development of effective anti-cancer therapy. This study examined specifically the expression of uPAR and TF because our group has developed imaging and treatment agents targeting these to cell membrane receptors [7–9]. uPAR and TF have consistently been associated with cancer in most types of solid carcinomas [10, 11]. In addition, EGFR expression was investigated because it is an established target for therapy in HNSCC. However, data on the utility of EGFR as target for imaging agents are lacking.

In head and neck squamous cell carcinoma (HNSCC), the role of Endothelial Growth Factor Receptor (EGFR) in cancer progression has been extensively investigated. Several studies found EGFR overexpression to be a negative prognostic factor for local control and survival outcome measures for tumors arising in different sub-sites in the upper aerodigastive tract. However, existing data with regard to the prognostic role of EGFR in OSSC is ambiguous [12]. The recent clinical introduction of anti-EGFR agents (i.e. Cetuximab) for treatment of advanced HNSCC has emphasized the potential of EGFR as a target for anti-cancer therapy [13]. Importantly, a clinical trial on EGFR-targeted intraoperative optical tumor imaging was recently published, and EGFR-directed PET-imaging has been demonstrated in preclinical studies [14, 15].

uPAR signaling stimulates pericellular proteolysis facilitating plasmin-mediated extracellular matrix (ECM) degradation and subsequent tumor cell migration and invasion. Because of abundant implications in the carcinogenesis of numerous types of cancer, uPAR has been regarded as a promising biomarker for targeted molecular imaging and therapy [16–18]. Also in OSCC the pathophysiological role of the plasminogen activator system has been investigated, and uPAR has been appointed a key role in process of local invasion in the interplay with other cancer-associated proteolytic systems and signaling pathways [19]. However, there is a need to further uncover the prognostic value of uPAR in OSCC and to explore the rational of uPAR-targeted strategies in this cancer entity.

A strong relation between cancer and hemostasis is generally accepted, and aberrant venous thromboembolism is a common manifestation in malignant disease, including HNSCC [20, 21]. TF is a transmembrane protein receptor and the principle initiator of the extrinsic coagulation cascade leading to fibrin formation after activation by its natural ligand factor VII. In addition TF activation has been associated with angiogenesis, tumor growth and invasion through regulation of the proteolytic cascade necessary for ECM degradation and tissue remodeling [22]. To our knowledge, TF expression in HNSCC has not been explored previously.

Accordingly, the aim of this study was to investigate the prognostic value and tumor expression patterns of EGFR, uPAR and TF in OSCC [23].

## Methods

### Patients

A cohort of 191 patients with primary OSCC, who underwent surgical tumor resection at the department of ORL – Head & Neck Surgery & Audiology at Rigshospitalet from 2000 to 2012, was retrospectively assembled. Inclusion criteria were primary OSCC in the mobile tongue or floor of mouth (FOM) with resection specimens available for immunohistochemical (IHC) analysis. Exclusion criteria were a previous history of HNSCC or radiotherapy to the head and neck region. Clinicopathological data were collected from medical records and pathology reports. All patients underwent clinical examination and radiological work-up and were staged at time of diagnosis according to the TNM classification by UICC, 7th edition [24]. Presence of regional nodal disease was determined based on pathology postoperatively (pN). A clear margin (>5 mm) was defined, according to the Royal College of Pathologists, as the absence of involved (<1 mm) or close margins (1–5 mm) on routine histology [25]. If intraoperative frozen section technique was applied, absence of tumors cells in these specimens also defined a clear margin. For survival analysis the last day for follow-up was 16th August 2016, and time of surgery, time of death of all causes and time of recurrence was recorded. The study was approved by the Ethical Committee of the Capital Region of Denmark (protocol H-2-2012-050).

### IHC staining

From formalin-fixed, paraffin-embedded tumor resection specimens, adjacent 4 μm sections were prepared. IHC staining was performed on a semi-automated auto-stainer (Benchmark Ultra, Ventana- Roche, CA, USA). All antibodies were applied in optimized dilutions previously determined using positive and negative control staining. Briefly, slides were deparaffinized and rehydrated using EZ prep solution (Ventana-Roche, CA, USA). Following monoclonal antibodies were used: Cytokeratin (CK) clone AE1/AE3 (1:200, DAKO, Glostrup, Denmark), EGFR (RTU, Ventana-Roche, CA, USA), mouse anti-human uPAR R2 (1:20.000, Finsen Laboratory, Copenhagen, Denmark) [26], TF #4509 (1:150, American Diagnostica Inc., Stamford, CT, USA). Antibody incubation time was 32 min. For EGFR, uPAR and TF and 24 min. For TF. Antigen retrieval for uPAR was done with protease K (Ventana-Roche, CA, USA) for 8 min followed by heating at 100 °C with cell conditioning 1 (CC1, Ventana-Roche, CA, USA) buffer for 16 min. For CK, TF and EGFR standard heat induced epitope retrieval (32 min, 100 °C) in CC1 buffer was used. IHC stainings were counterstained with hematoxylin. In addition, a section stained for hematoxylin-eosin (HE) of each case was prepared.

### Histology scoring

All cases were reviewed and scored by two specialized head and neck pathologists (GL and KK) blinded to the clinicopathological data. For each case, the presence of tumor and extent of the tumor-compartment in relation to surrounding normal tissue was evaluated on HE and CK sections. The expression of uPAR, TF and EFGR was scored for intensity (I-score) and proportion of IHC reactivity within the tumor compartment (P-score). Both the I-score and the P-score were based on a 4-point system: 0–3+ (none, weak, moderate strong and 0–10%, 11–50%, 51–75%, 76–100%, respectively). For the I- and P-score IHC reactivity was not subdivided into expression on tumor cells and stromal cells, but was evaluated together, to represent reaction in the whole tumor-compartment. To combine information of intensity and proportion, a combined score (PI-score) was formed by addition of the I- and P-score as proposed by Allred and colleges [27]. The PI-score formed a 7-point system with a semi-quantitative scale from 0 to 6. In addition, sections were evaluated for homogeneous IHC expression within the tumor-compartment (yes/no), and IHC positivity in dysplastic epithelium if present (yes/no). Because this paper investigated two separate research questions, expression patterns in relation to biomarker utility for targeted strategies and biomarker prognostication, respectively, the PI-score was dichotomized in two ways. For the evaluation of expression rate of uPAR, TF and EGFR, a tumor was considered positive if the PI-score was >1. A dichotomization in positive vs. negative was chosen because the positive expression rate across a cohort of patients is a key figure to determine the utility of a biomarker for targeted imaging. In the analysis of the prognostic value of uPAR, TF and EGFR, the cut-off values to separate low and high expression was defined as IPS < 6 for uPAR and EGFR and IPS < 3 for TF based on the distribution of the score for each biomarker. Histology sections were scanned using Axio Scan.Z1 (Carl Zeiss, Jena, Germany) to create digital images.

### Statistical analysis

Associations between biomarker expression and clinicopathological variables were analyzed by Pearson’s chi-square test or Fisher’s exact test for small numbers. Age differences were investigated using the two-sample t-test. Overall survival (OS) was defined as time from primary surgery to death due to any cause, and disease-free survival (DFS) was defined as time from primary surgery to cancer relapse or death by any cause. Associations between biomarker expressions and survival outcomes were visualized in Kaplan-Meier plots using the log-rank test to assess significance of differences. Also, the Kaplan-Meier method was used to estimate 5-year survival estimates. Hazard ratios were estimated in univariate and multivariate Cox proportional hazards model adjusting for gender, age, tobacco history, T-site, margin status, T-stage, N-stage, extracapsular spread (ECS), TNM stage and tumor differentiation. A *p*-value <0.05 was considered statistically significant. All data analysis was performed in the SAS software package (SAS Institute Inc., version 6.1, USA).

## Results

The retrospective cohort of 191 patients with primary OSCC had a male predominance (66%), the median age at time of surgery was 59 years (range: 23–89 years). The median follow-up was 5.1 years (range: 0.1–15.9 years). Demographics and the clinicopathological variables are listed in Table 1. Overall, the anatomical location of the tumors was distributed almost equally as 98 FOM tumors and 93 tongue tumors. The majority of the patients (86%) presented with early stage disease (S1-S2) and most tumors (90%) were well differentiated (G1-G2). 31% were diagnosed with primary regional nodal disease and 51 patients (27%) had post-treatment relapse. For the entire cohort the 5-year OS and DFS was 51% and 41%, respectively.

**Table 1:** Correlation analysis of clinicopathological findings and biomarker expression

Variable		Total N (%)	Low uPAR N (%)	High uPAR N (%)	*P*-value	Low TF N (%)	High TF N (%)	*P*-value	Low EGFR N (%)	High EGFR N (%)	*P*-value
Gender	Men	126 (66)	94 (69)	32 (59)		73 (63)	53 (70)		71 (62)	55 (71)	
	Women	65 (34)	43 (31)	22 (41)	0.219	42 (37)	23 (30)	0.372	43 (38)	22 (29)	0.191
Age^b^	−	−	−	−	0.121	−	−	0.059	−	−	0.190
Tobacco	Never	32 (17)	22 (16)	10 (19)		20 (17)	12 (16)		20 (18)	12 (16)	
	Ever	159 (83)	115 (84)	44 (81)	0.682	95 (83)	64 (84)	0.772	94 (82)	65 (84)	0.722
	Low^c^	46 (24)	32 (23)	14 (26)		30 (26)	16 (21)		27 (24)	19 (25)	
	High	145 (76)	105 (77)	40 (74)	0.709	85 (74)	60 (79)	0.426	87 (76)	58 (75)	0.875
T-site	Tongue	93 (49)	69 (50)	24 (44)		48 (42)	45 (59)		53 (46)	40 (52)	
	FOM	98 (51)	68 (50)	30 (56)	0.461	67 (58)	31 (41)	**0.018**	61 (54)	37 (48)	0.459
Relapse	Yes	51 (27)	38 (28)	13 (24)		23 (20)	28 (37)		33 (29)	18 (23)	
	No	140 (73)	99 (72)	41 (76)	0.455	92 (80)	48 (63)	**0.010**	81 (71)	59 (77)	0.393
	T-site	24 (38)	19 (44)	5 (25)		13 (39)	11 (37)		7 (39)	17 (38)	
	N-site	27 (43)	19 (44)	8 (40)		10 (30)	17 (57)		9 (50)	18 (40)	
	Both	12 (19)	5 (12)	7 (35)	0.071	10 (30)	2 (7)	**0.028**	2 (11)	10 (22)	0.566
Margins	Negative	107 (56)	77 (56)	30 (56)		66 (57)	41 (54)		63 (55)	44 (57)	
	Positive	84 (44)	60 (44)	24 (44)	0.935	49 (43)	35 (46)	0.639	51 (45)	33 (43)	0.798
T-stage	T1-T2	164 (86)	119 (87)	45 (83)		96 (83)	68 (89)		97 (85)	67 (87)	
	T3-T4	27 (14)	18 (13)	9 (17)	0.529	19 (17)	8 (11)	0.244	17 (15)	10 (13)	0.708
N-stage	N0	131 (69)	98 (72)	33 (61)		80 (70)	51 (67)		77 (68)	54 (70)	
	N+	60 (31)	39 (28)	21 (39)	0.162	35 (30)	25 (33)	0.720	37 (32)	23 (30)	0.706
ECS	Yes	16 (8)	10 (7)	6 (11)		10 (9)	6 (8)		11 (10)	5 (6)	
	No	175 (92)	127 (93)	48 (89)	0.395^a^	105 (91)	70 (92)	0.845	103 (90)	72 (94)	0.440
TNM Stage	S1-S2	136 (71)	104 (76)	32 (59)		81 (70)	55 (72)		82 (72)	54 (70)	
	S3-S4	55 (29)	33 (24)	22 (41)	**0.022**	34 (30)	21 (28)	0.773	32 (28)	23 (30)	0.788
Grade	G1-G2	171 (90)	121 (88)	50 (93)		105 (91)	66 (87)		103 (90)	68 (88)	
	G3-G4	20 (11)	16 (12)	4 (7)	0.385	10 (9)	10 (13)	0.324	11 (10)	9 (12)	0.652
	G1	37 (19)	26 (19)	11 (20)		23 (20)	14 (18)		24 (21)	13 (17)	
	G2	134 (70)	95 (69)	39 (72)		82 (71)	52 (68)		79 (69)	55 (71)	
	G3	20 (11)	16 (12)	4 (7)		10 (9)	10 (13)		11 (10)	9 (12)	
	G4	0(0)	0 (0)	0 (0)	0.684	0 (0)	0 (0)	0.612	0 (0)	0 (0)	0.732

Chi-square test. ^a^Fisher’s exact test used because of small numbers. ^b^Age was treated as a continuous variable and correlations was calculated with a Student t-test. ^c^Low defined as <10 pack years. Significant *p*-values of 0.05 or less are presented in bold

### Histopathological biomarker expression patterns

**Fig. 1:**
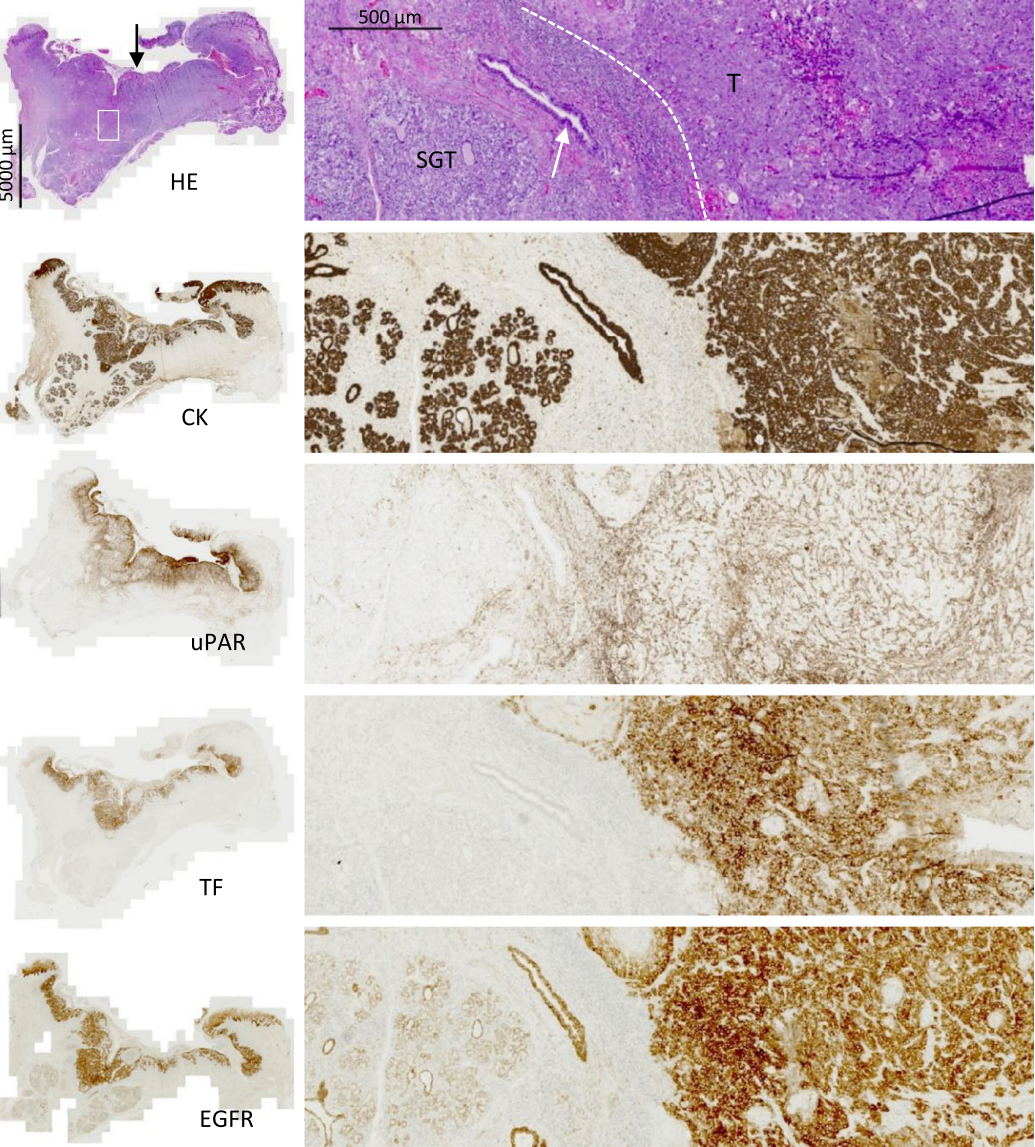
Patterns of expression of uPAR, TF and EGFR. Adjacent tissue sections from a T1 FOM tumor (T). Black arrow indicates the epithelial lining in the oral cavity. White square shows the location of the enlarged region of interest presented to the right. Dotted white line shows the invasive front of the tumor. A large collecting salivary duct (white arrow) and salivary gland tissue (SGT) located adjacent to the tumor border. EGFR expression is noted on neoplastic cells as well as in the epithelium of salivary gland tissue

**Fig. 2:**
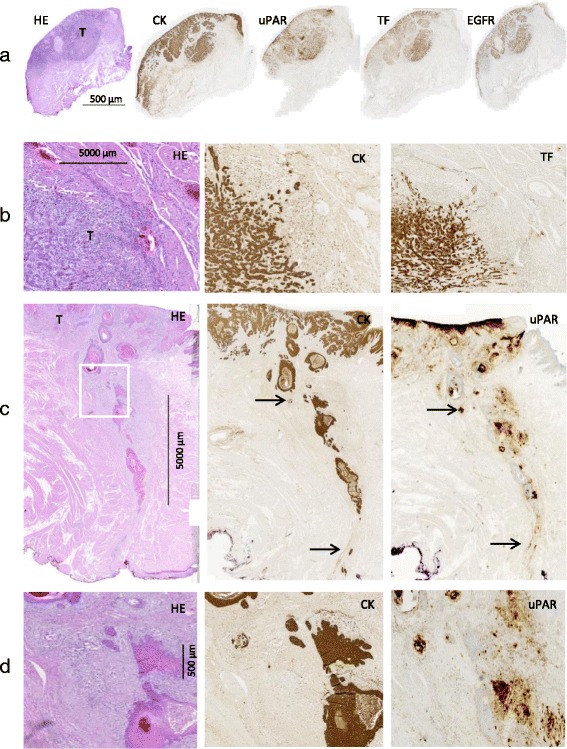
Selected features of biomarker expression**. a** T2 tongue SCC (T). Expression of uPAR, TF and EGFR confined to the tumor compartment. **b** An example of strong TF expression on the neoplastic cells at the invasive front of a tongue SCC (T). **c** An example of a tongue SCC T) invading deeply into the underlying stroma. Black arrows indicate expression of CK and uPAR on small tumor cell groups and the white square shows the location of the enlarged region of interest depicted in panel (**d**)

Based on the examination of the immunohistochemical staining in resection specimens, uPAR and TF were found to be highly tumor-specific, with enhanced expression within the tumor compartment, and absent or very limited expression in the normal tissues surrounding the tumors (Figs. 1 and 2). EGFR was less tumor-specific due to regular expression in normal tissues. The overall positive expression rate of uPAR, TF and EGFR was 95%, 58% and 98%, respectively. High expression of uPAR, TF and EGFR was observed in 28%, 39% and 40% of the tumors, respectively (Table 2).

**Table 2:** Expression patterns of uPAR, TF and EGFR in OSCC

Variable		uPARN (%)	TFN (%)	EGFRN (%)
Positive biomarker expression	Yes	182 (95)	110 (58)	188 (98)
	No	9 (5)	81 (42)	3 (2)
Homogeneous expression in the tumor compartment	Yes	107 (56)	50 (26)	163 (85)
	No	84 (44)	141 (74)	28 (15)
Expression in dysplastic epithelium^a^	Yes	4 (4)	16 (16)	92 (94)
	No	94 (96)	82 (84)	6 (6)

^a^Dysplastic epithelium was present in 98 out of 191 tumor samples

uPAR was expressed on neoplastic cells as well as on fibroblasts and inflammatory cells in the tumor compartment. In addition, stromal expression of uPAR was also the predominant pattern. The same pattern was observed in isolated clusters of neoplastic cells invading into the stroma (Fig. 2). Also, in the majority of the tumors, strong uPAR expression was found in a narrow and well demarcated peritumoral reactive zone at the invasive front. uPAR expression in dysplastic epithelium adjacent to the carcinoma was very limited (4.1%, Table 2). In most specimens, solitary uPAR-positive neutrophils were observed in the tumor compartment as well as in normal tissues. In two cases, strong uPAR staining was noted on macrophages and neutrophils in an abscess located outside the tumor compartment.

Within the tumor compartment, TF was predominantly expressed in a heterogeneous pattern on tumor cells, and intense staining was generally noted at the invasive edge of the tumors (Fig. 2). TF expression in dysplastic epithelium was noted in some cases (16.3%) and the basal cells of normal epithelium showed weak TF-positivity in all cases. Generally, a weak staining of TF was observed in the collecting ducts in salivary gland tissue outside the tumor compartment.

EGFR was found to have highly homogeneous expression on neoplastic cells in the majority of the tumors (Table 2). Moreover, EGFR expression outside the tumor compartment was observed in the normal epithelium in all cases. In specimens containing dysplasia, EGFR was expressed in the dysplastic epithelium in 93.9% of the cases. In adjacent normal salivary gland tissue, regular EGFR expression was seen on the epithelial cells (Fig. 1).

### Correlation analysis of biomarker expression and clinicopathological parameters

Associations between biomarker expression and clinicopathological variables are summarized in Table 1. High uPAR expression was significantly correlated with an advanced TNM stage (*p* = 0.022). High TF expression was significantly associated with tumor location in the tongue (*p* = 0.018) and relapse of disease (*p* = 0.001). No other significant associations between the three biomarkers and clinicopathological variables were observed.

### Survival analysis

**Fig. 3:**
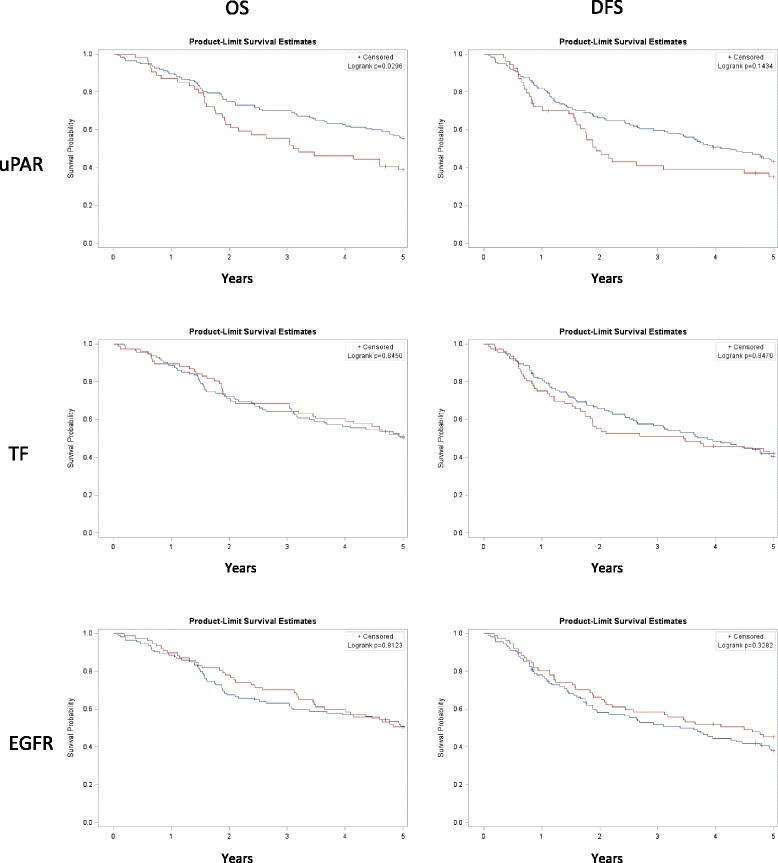
Kaplan Meier curves showing 5-year OS and DFS for expression of uPAR, TF and EGFR**.** Red line: High expression, blue line: low expression. Difference in survival of high compared to low expression was calculated by the log rank test

Univariate and multivariate survival analysis using the Cox proportional hazard model for OS and DFS with respect to variables are summarized in Table 3. In OS and DFS 126 and 177 events were recorded, respectively. Kaplan Meier curves combined with log rank analysis for differences showed a significant association between high uPAR expression in tumors and OS but not DFS, and no significant correlations was found for TF and EGFR (Fig. 3). The 5-year OS was 55.5% for low uPAR expression and 39.1% for high uPAR expression (*p* = 0.030). High uPAR expression showed a significant negative association with OS in the univariate analysis (*p* = 0.031, HR = 1.595 (95%CI 1.044–2.439)) but significance of this association was not retained in the multivariate analysis (*p* = 0.128, HR =1.435 (95%CI 0.901–2.287)). uPAR expression did not reach statistical significance in DFS. For TF and EGFR no significant association with survival outcome was detected. To investigate a possible prognostic value of uPAR, TF and EGFR in early low-risk disease, univariate and multivariate analysis was also performed for the subgroups S1-S2 (*n* = 136), T1-T2 (*n* = 164) and G1-G2 (*N* = 171). High uPAR expression reached a significant association with OS only in the univariate analysis in all three subgroups but not in the multivariate analysis. However, in the sub-group of well differentiated tumors (G1-G2) a borderline significant association was detected in multivariate analysis (*p* = 0.051, HR = 1.618 (95%CI 0.997–2.625)).

**Table 3:** Uni- and multivariate analyses using Cox proportional hazards model for OS and DFS in relation to clinocopathological variables and biomarker expression for in 191 OSCC patients

		Overall Survival (OS)						Disease Free Survival (DFS)					
		Univariate analysis			Multivariate analysis			Univariate analysis			Multivariate analysis		
Variable		*p*-value	HR	95% CI	*p*-value	HR	95% CI	*p*-value	HR	95% CI	*p*-Value	HR	95% CI
Gender	Men	0.745	1.075	0.696–1.658	0.260	1.320	0.814–2.140	0.576	1.121	0.751–1.673	0.147	1.386	0.891–2.155
	Women												
Age^b^	−	**0.027**	1.019	1.002–1.037	**0.002**	1.034	1.012–1.056	**0.017**	1.019	1.003–1.035	**0.005**	1.027	1.008–1.047
Tobacco	Low	0.533	1.167	0.718–1.896	0.873	1.047	0.594–1.847	0.991	0.997	0.650–1.531	0.953	0.985	0.592–1.638
	High												
T-site	Tongue	0.355	1.211	0.807–1.818	0.786	0.935	0.577–1.516	0.885	1.028	0.708–1.491	0.200	0.743	0.472–1.170
	FOM												
Margins	Negative	**<0.000**	2.385	1.580–3.600	**0.009**	1.788	1.788–2.767	**<0.000**	2.457	1.681–3.590	**0.000**	2.126	1.418–3.189
	Positive												
T-stage	T1-T2	**0.001**	2.326	1.417–3.818	0.335	1.346	0.736–2.464	**0.005**	1.987	1.232–3.204	0.364	1.305	0.735–2.317
	T3-T4												
N-stage	N0	**<0.000**	2.492	1.692–3.755	**0.046**	1.885	1.011–3.515	**<0.000**	2.220	1.512–3.259	**0.047**	1.814	1.008–3.263
	N+												
ECS	No	**0.000**	0.310	0.172–0.560	0.118	0.551	0.262–1.262	**<0.000**	0.293	0.166–0.518	0.059	0.497	0.241–1.028
	Yes												
TNM stage	S1-S2	**<0.000**	2.606	1.721–3.945	0.889	1.052	0.515–2.149	**0.000**	2.115	1.428–3.133	0.930	0.971	0.501–1.882
	S3-S4												
Grade	G1-G2	0.104	0.606	0.330–1.109	0.084^a^	0.676	0.353–1.293	0.084	1.639	0.935–2.874	0.115^a^	0.613	0.340–1.104
	G3-G4												
	G2												
	G3		0.672	0.364–1.241					0.680	0.385–1.199			
	G4	0.063	0.394	0.180–0.864		0.084	0.214–1.103	0.039	0.399	0.194–0.819		0.455	0.215–0.964
Radiotherapy	Yes	**0.005**	1.776	1.185–2.663	0.114	1.451	0.914–2.303	0.096	1.375	0.945–1.998	0.201	1.318	0.863–2.014
	No												
uPAR	High	**0.031**	1.595	1.044–2.439	0.128	1.435	0.901–2.287	0.145	1.351	0.902–2.025	0.326	1.246	0.803–1.931
	Low												
TF	High	0.846	0.960	0.634–1.452	0.977	0.993	0.629–1.569	0.846	1.038	0.710–1.519	0.445	1.179	0.773–1.798
	Low												
EGFR	High	0.813	0.952	0.630–1.437	0.922	1.023	0.654–1.599	0.329	0.826	0.563–1.212	0.219	0.766	0.501–1.172
	Low												

Significant *p*-values of 0.05 or less are highlighted in bold. ^a^Grade had multilevel specification and an overall *p*-value for the group-covariate was calculated in the multivariate analysis. ^b^Age entered the analyses as a continuous variable

As expected, high T-stage, high disease stage, involved or close resection margins, primary nodal metastasis and presence of ECS was associated with poor outcome in OS and DFS in univariate analysis. Only margin status and N-stage retained significance in multivariate analysis. Also increasing age was associated with reduced OS and DFS in univariate and multivariate analysis.

## Discussion

This study provides novel data compared to existing literature because it was based on IHC biomarker expression in whole tumor resection specimens of OSCC and not only biopsy specimens. Resection specimens containing both the tumor compartment and the adjacent resection margin of healthy tissue is a prerequisite to accurately examine the utility of a biomarker for tumor-specific molecular targeting. Also, resection specimens allows to assess the heterogeneity of the biomarker expression across the tumor compartment as opposed to studies based on biopsies that only sample a very small fraction of the tumor. To our knowledge, this type of study of uPAR and TF expression in OSCC has not previously been reported.

uPAR and TF both showed an enhanced expression specifically confined to the tumor compartment with very limited expression in the normal tissues surrounding the neoplasm. In contrast, EGFR lacked a tumor-specific expression pattern and therefore the ability to distinguish between malignant and normal tissues. Further, the positive expression rate of uPAR and TF was high, which implies, that a substantial part of OSCC patients should be regarded as candidates for imaging and/or therapy directed against either uPAR or TF. Especially uPAR was found to have a highly tumor-specific pattern, and also with very limited expression in both normal and dysplastic epithelium around the epithelial tumor lesion. In comparison, EGFR exhibited staining of both normal and dysplastic epithelium in most cases, and importantly also a general EGFR expression in salivary gland tissue outside the tumor compartment was seen. A prerequisite to develop highly accurate targeted imaging is that the molecular target exhibits very low expression in normal tissues bordering the tumor to create a high tumor-to-normal tissue ratio. Targeted optical-guided surgery is currently being clinically translated, and this imaging modality will allow intraoperative real-time assessment of resection margins in order to ensure complete removal of tumors. Further, our group and others have recently presented preclinical data derived from animal models of oral cancer, showing that detection of subclinical disease by use of targeted fluorescent probes is possible [28, 29]. However, because an optical imaging signal has a low energy with a limited range in intensity, target binding of an imaging agent outside the tumor compartment in normal tissues would potentially have substantial influence on the ability to detect a reliable tumor-specific signal to guide a tumor resection. Accordingly, in the data from the recently published first phase 1 trial of targeted optical imaging in HNSCC, using the optical agent cetuximab-IRDye800 directed against EGFR, extratumoral signal uptake in normal epithelium and salivary gland tissue in tissue sections was reported [30].

Direct comparison of the positive expression rates of uPAR, TF and EGFR in this study and previous studies is not possible because of difference in scoring systems used and because of different cut-off values to determine positive and negative expression. Three studies investigated expression of uPAR in OSCC and reported a positive expression rate in the range of 39–100% [31–33]. Positive EGFR expression in OSCC was in the range of 60–100% in previous studies [34–37]. We found a positive expression rate of 58% for TF, but the expression of TF in OSCC has not previously been investigated. Chen et al. found a TF immunopositivity of 91% in esophageal cancer [38].

To select potential biomarkers for targeted imaging, van Oosten et al. suggested a selection criteria tool identifying seven factors on order of importance: (1) Extracellular receptor location, (2) diffuse enhanced target expression in tumor compartment, (3) high tumor-to-normal tissue target expression, (4) high expression-rate in patients, (5) previous successful targeted imaging in vivo, (6) enzymatic activity of the receptor and (7) target receptor internalization [39]. In relation to the results of the present study, both uPAR, TF and EGFR fulfills the factors 1–5, which underlines the relevance of these receptors for targeted tumor imaging in OSCC. In addition, uPAR is also reported to exhibit enzymatic activity in the tumor microenvironment and internalization to the intracellular space upon ligand-binding, which makes this receptor especially suited for targeting by imaging agents [11].

In the correlation analysis, high TF expression was associated with relapse of disease. However, TF expression did not show significant impact on OS or DFS in the survival analysis, which has been reported in colorectal, breast and esophageal cancer [38, 40, 41]. We found high uPAR expression to be associated with late TNM stage disease (S3-S4). However, no significant association was reached between uPAR and N- or T-stage analyzed separately. In a study of 115 patients with OSCC, Magnusson et al. reported that low expression of uPAR was correlated with reduced disease specific death only in patients with stage 1 (S1) disease [42]. Bacchiochi et al. analyzed the prognostic value of uPAR in 189 patients with OSCC and found enhanced uPAR expression to be associated with increasing tumor cell differentiation, and that low uPAR expression only was associated with increased OS in well differentiated tumors [31]. Our study confirms the findings in the latter study, as high uPAR expression only for the sub-group of well differentiated tumors was associated negatively with OS in the univariate analysis and reached borderline significance in the multivariate analysis. In two studies from the same Japanese group, containing 34 and 54 OSCC patients, respectively, high uPAR expression was associated with an aggressive mode of invasion [43, 44]. Unfortunately, our study did not include the pattern of invasion (cohesive vs. non-cohesive invasive tumor front) as a histopathological variable, but further research in the relation between local tumor aggressiveness and uPAR activity in OSCC is warranted.

In the survival analysis in the present study, enhanced uPAR expression was associated with a significant reduction in OS only, while no significant associations between expression of TF or EGFR and survival outcome could be demonstrated. Accordingly, our data supports findings in previous studies, that uPAR expression seems to be a prognostic factor for survival outcome in OSCC [31, 42, 44]. However, we did not find uPAR to be an independent prognostic factor in multivariate analysis. Also, uPAR was associated with advanced TNM stage. Therefore, uPAR expression could also be a surrogate for advanced stage of disease as well as comorbidity. A larger sample size would be able to clarify the meaning of these findings.

Interestingly, our study is the first to show that uPAR had impact on survival outcome across an entire cohort of patients, and not only in a subgroup analysis of patients defined by a specific clinicopathological variable [31, 42] or a combination of biomarker expressions [44]. We did not find any significant association between EGFR expression and survival outcome, which is consistent with several previous studies, although some studies have associated enhanced EGFR expression with poor clinical outcome [12]. Of note, a limitation of this study was a relatively small sample size with a limited number of outcome events, which may have affected the ability to detect significant correlations between biomarker expression and survival. In addition, assessment of biomarker expression based on immunohistochemistry has an inherent limited accuracy due to intra-observer variability and the design of the scoring systems used.

Consistent with a study of Lindberg et al., we also found that uPAR predominantly was expressed on stromal cells of the tumor compartment [33]. Similar findings have been reported in colorectal and esophageal cancer [45, 46]. Stroma-rich tumors, like OSCC, may be less sensitive to targeted therapy, if the target only is expressed by tumor cells, because of reduced target density in the tumor compartment. Therefore, uPAR expression on both tumor cells and tumor-associated stromal cells may provide uniform target availability in the entire tumor volume. This expression pattern may be a particular advantage in order to achieve high efficacy of uPAR-targeted intervention and therapy.

Existing data indicate, that elevated uPAR expression in OSCC and several other types of cancer within the tumor seem to predict a more aggressive phenotype that carries reduced survival outcome [18]. Therefore uPAR may have a role to play as a reliable prognostic biomarker in future personalized management of OSCC. Based on the favorable properties of uPAR as an imaging target, a clinical phase 2 trial of preoperative uPAR-PET/CT imaging in patients with oral or oropharyngeal SCC is currently being conducted in our institution (NCT02960724). uPAR-PET imaging may provide a non-invasive quantitative assessment of the uPAR expression in the entire volume of individual tumors, thereby surpassing the inherent problems related to surgical biopsies and risk of sampling error due to tumor heterogeneity.

## Conclusions

Our results showed that both uPAR and TF had high positive expression rates and tumor-specific expression patterns while EGFR also had regular expression in normal tissues. These findings may suggest that uPAR and TF could potentially be attractive targets for molecular imaging and targeted therapy in OSCC.

High uPAR expression was significantly associated with reduced survival outcome. Accordingly, uPAR seems to be a potential prognostic biomarker in OSCC, which may have applications for risk-stratification and treatment planning.

## Abbreviations

95%CI: 95% Confidence Interval
CC1: Cell conditioning 1
DFS: Disease-free Survival
EGFR: Epidermal Growth Factor Receptor
FOM: Floor of mouth
HNSCC: Head & Neck Squamous Cell Carcinoma
IHC: Immunohistochemistry
OS: Overall Survival
OSCC: Oral Squamous Cell Carcinoma
TF: Tissue Factor
UPAR: Urokinase-type Plasminogen Activator Receptor
